# Media temperature control: a potentially important quality control parameter in human oocyte vitrification

**DOI:** 10.1530/RAF-26-0003

**Published:** 2026-05-15

**Authors:** Munevver Serdarogullari, Zafer Atayurt, Georges Raad, Yaren Yilancilar, Omar Ammar, Zalihe Yarkiner, Beril Yuksel, Marcos Meseguer, George Liperis

**Affiliations:** ^1^ Ventus IVF Center, Embryology Laboratory, Nicosia, Cyprus; ^2^ Department of Histology and Embryology, Faculty of Medicine Cyprus International University, Northern Cyprus, Turkey; ^3^ Embryology Laboratory, Nicosia, Cyprus; ^4^ Ventus IVF Center, Nicosia, Cyprus; ^5^ Faculty of Medicine and Medical Sciences, Holy Spirit University of Kaslik, Jounieh, Lebanon; ^6^ Al Hadi Laboratory and Medical Centre, Beirut, Lebanon; ^7^ Medicine, Beirut, Lebanon; ^8^ Department of Obstetrics and Gynaecology, College of Medicine, University of Anbar, Ramadi, Iraq; ^9^ Department of Basic Sciences and Humanities, Faculty of Arts and Sciences, Cyprus International University, Northern Cyprus, Turkey; ^10^ Basic Sciences and Humanities, Nicosia, Cyprus; ^11^ Department of Obstetrics and Gynecology – Girne American University, Girne, Cyprus; ^12^ IVIRMA Global Research Alliance, IVI Foundation, Instituto de Investigación Sanitaria La Fe IIS La Fe, Valencia, Spain; ^13^ IVIRMA Global Research Alliance, IVIRMA Valencia, Valencia, Spain; ^14^ Research, Valencia, Spain; ^15^ University of Sydney, New South Wales, Australia; ^16^ Embryorigin Fertility Centre, Larnaca, Cyprus

**Keywords:** oocyte vitrification, embryo development, temperature control, quality control, pronuclear arrest

## Abstract

**Abstract:**

The purpose of this study was to evaluate the role of controlling media temperature during human oocyte vitrification. This retrospective study compared fertilisation rates, cleavage-stage embryo development, and blastulation between two separate periods defined by uncontrolled versus controlled media temperature during oocyte vitrification. A retrospective observational study was conducted at Ventus IVF Center, Cyprus, evaluating the effect of vitrification solution temperature during vitrification on oocyte survival and embryological outcomes. Oocytes were vitrified either under uncontrolled ambient conditions (*n* = 87 oocytes) or with temperature control (*n* = 83 oocytes), with the experimental groups assessed asynchronously. Vitrification and equilibration solution temperatures were recorded using a calibrated UNI-T UT322 thermometer. There were no significant differences in baseline characteristics of oocyte donors between the controlled and uncontrolled temperature groups (*P* > 0.05). During vitrification, vitrification media temperature was significantly higher in the controlled group (23.85 ± 0.43°C) compared to the uncontrolled group (19.65 ± 0.43°C; *P* < 0.001). No significant differences were observed in oocyte survival post-warming (82/83 vs 80/87; *P* = 0.08) or fertilisation rates (69/82 vs 70/87; *P* = 0.67) between groups. Compared to the controlled group, the uncontrolled group showed markedly higher developmental arrest at the pronuclear (10/70 vs 0/69; *P* < 0.001), post-pronuclear (20/70 vs 0/69; *P* < 0.001), and cleavage (35/70 vs 0/69; *P* < 0.001) stages, and significantly reduced blastocyst formation (5/70 vs 56/69; *P* < 0.001). Maintaining vitrification solution temperature rather than relying on ambient conditions significantly improves oocyte vitrification efficiency and pre-implantation embryo development parameters.

**Lay summary:**

Freezing human eggs is widely used in fertility treatment and fertility preservation, but small laboratory factors can strongly influence success. This study investigated whether controlling the temperature of the freezing solutions during egg freezing into a glass-like state affects how well eggs survive and develop into embryos. Eggs from donor cycles were frozen either under room conditions or by controlling the freezing solution temperature at a stable temperature. Although egg survival and fertilisation rates were similar in both groups, eggs frozen without temperature control showed very high rates of early embryo development failure and produced very few embryos following a five-day culture. In contrast, eggs frozen with controlled solution temperatures developed into embryos following the five-day culture far more often. These findings show that maintaining the temperature of freezing solutions is a critical but often overlooked laboratory quality control factor and that simple temperature control measures could significantly improve the success of egg freezing in fertility clinics.

**Graphical Abstract:**

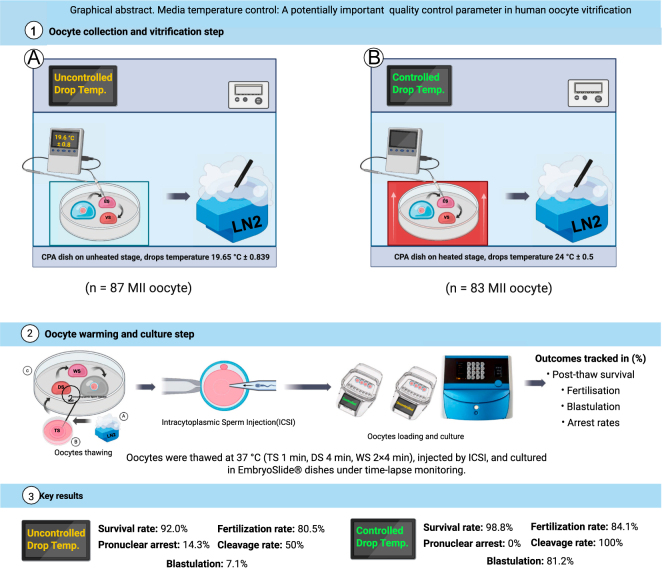

## Introduction

Vitrification has emerged as the leading method for oocyte cryopreservation, marking a major advancement in medically assisted reproduction (MAR), as demonstrated by its clinical effectiveness, safety, and widespread adoption in fertility preservation and elective oocyte freezing worldwide (Cobo *et al.* 2011, Argyle *et al.* 2016, Ethics Committee of the American Society for Reproductive Medicine. Electronic address: asrm@asrm.org and Ethics Committee of the American Society for Reproductive Medicine 2018, Johnston *et al.* 2021, Cascante *et al.* 2023). Its established role in fertility preservation for medical and non-medical indications, as well as oocyte donation programmes, particularly through decoupling donor–recipient cycle synchronisation, further supports its position as the gold standard (Cobo *et al.* 2011, Martínez-Burgos *et al.* 2011). However, given the widespread use of oocyte vitrification, evidence-based modifications to current protocols are essential to optimise outcomes.

Human oocytes are large cells with high water content and a distinct cytoskeleton (Segovia *et al.* 2017). During *in vitro* handling and culture, even a short drop from the standard 37°C culture temperature has been associated with effects on the meiotic spindle (Wang *et al.* 2001*a*,*b*). Spindle disorganisation begins after ∼10 min at 33–34°C (Zenzes *et al.* 2001) and becomes complete within minutes at 25°C (Larman *et al.* 2007), and exposure to 0°C has been shown to cause loss of spindle integrity and chromosomal misalignment (Koutlaki *et al.* 2006). Nonetheless, such effects have been observed to be transient, with the spindle being a dynamic structure capable of depolymerisation and repolymerisation (Sharma *et al.* 2013).

The process of vitrification entails exposing oocytes to cryoprotectant (CPA) solutions followed by rapid cooling, turning water into a glass-like structure and avoiding hazardous intracellular ice formation (Martinez-Rodero *et al.* 2025). The method of vitrification works effectively only if all factors stay in balance (Chang *et al.* 2024, Martinez-Rodero *et al.* 2025). If CPA exposure or concentration is too high, chemical toxicity and sudden water movement can create cryoinjury to the oocyte (Chang *et al.* 2024, Martinez-Rodero *et al.* 2025).

Temperature has a pivotal role in retaining the balance (Elder & Dale 2020). Handling at warmer conditions accelerates CPA diffusion into and out of the oocyte, so the oocyte spends less time in potentially toxic solutions (Elder & Dale 2020). Temperature loss is the first link in the chain of events leading to oocyte damage with this damage possibly remaining unnoticed until pre-implantation embryo development (Elder & Dale 2020). Vitrification commercial kit manufacturers suggest working at a stable room temperature. In practice, however, several parameters can affect the temperature at which oocytes are exposed while handling within the CPA solutions. These can include, among others, open dishes (without a lid) on an unheated laminar flow hood and laminar flow hood air flow speed that can cause cooling effects, dish material, and air-conditioning (Sjoblom & Liperis 2019, Elder & Dale 2020). These conditions can create substantial differences even in laboratories that apply the same vitrification protocols and in accordance with vitrification media manufacturers’ instructions (Medicine 2012). Among these conditions, temperature control during vitrification has traditionally focused on cooling and warming rates, with less attention given to the temperature of the vitrification solutions during handling and processing (Stachecki 2024).

This retrospective observational study was conducted in a single centre and covered two separate periods. During the first period, oocyte vitrification was performed according to the manufacturer’s instructions, with active monitoring of vitrification drop temperature but no control of the vitrification solution temperature and absence of maintenance within a designated temperature range (Medicine 2012, Stachecki 2024). During this period, vitrification was conducted under routine ambient laboratory conditions with no interventions.

This practice coincided with a noticeable decline in embryo developmental outcomes post-warming compared to the defined laboratory key-performance-indicators (KPIs) as established by the Vienna Consensus (‘The Vienna Consensus’ 2017), raising concerns about the potential impact of vitrification solution temperature, an aspect that had not previously been addressed as a critical quality control parameter. As part of troubleshooting, corrective measures were implemented to ensure strict temperature control of the vitrification solutions in subsequent procedures which consisted of the second period.

Different donors contributed to the two study groups due to the troubleshooting nature of this observational study. The study was initiated after observing suboptimal outcomes under routine laboratory conditions. It was, therefore, not possible to prospectively allocate sibling oocytes from the same donors to both groups. In addition, clinical and logistical constraints, as well as restrictions related to donor availability over different time frames, precluded recruitment of the same donors for both experimental conditions. This design reflects real-world laboratory practice while allowing assessment of the impact of temperature control on vitrification outcomes.

This study aimed to summarise the results of these two separate periods and study groups comparing embryological outcomes following oocyte warming, fertilisation, and embryo development to the blastocyst stage. By analysing survival rates and developmental competence, the aim was to identify if vitrification solution drop temperature is an important quality control parameter that requires control.

## Materials and methods

### Study design and ethical considerations

This is a retrospective and asynchronous observational study conducted at Ventus IVF Center, a newly established fertility centre in Cyprus, between March 2024 and February 2025. This study was approved by the Cyprus International University Scientific Research and Publication Ethics Committee. The clinic implemented a broad internal KPI monitoring system to build a reference dataset and ensure quality, with temperature routinely measured as an environmental indicator. Temperature measurements included ambient room temperature, all surface temperatures during handling, culture temperatures, and solution temperatures, including vitrification solution drop temperature. Surface, solution, and room temperatures were monitored using the UNI-T UT322 thermometer (Uni-Trend Technology Co., Ltd, China), which was validated against an NIST-traceable mercury-calibrated thermometer. The Vermox thermometer was equipped with a T-type thermocouple as the drop sensor and a PT100 RTD as the surface sensor. The UNI-T thermometer used a K-type thermocouple as the drop sensor, and temperature measurements were recorded at the frequency specified by the manufacturer until the temperature stabilised and no longer fluctuated.

During all stages of *in vitro* handling, including oocyte and embryo warming, denudation, fertilisation check, as well as ICSI, solution temperature was measured and documented using the validated thermometer and sensor. Measurements included both the solution drop temperature and the corresponding surface temperature (±SD), and adjustments to the surface temperature were made where necessary to ensure control of handling temperature at 37.0 ± 0.2°C. During oocyte and embryo culture within the incubators, temperature was monitored continuously using temperature data loggers and sensors to record temperature over time, providing real-time data and historical records for analysis integrated with automated alarms to ensure optimal incubator conditions and signal any deviations for immediate intervention.

Prior to allocating donor oocytes to recipients, the efficiency of the oocyte cryopreservation programme for donors for which oocytes were frozen between March 2024 and August 2024 was tested. During this period, while vitrification solution temperature was monitored, there was no control of the temperature within a designated range, as this was not in the list of recommendations by the vitrification media manufacturer. Donor oocytes were warmed and fertilised with donor sperm. The outcomes in terms of oocyte survival and subsequent embryo development were recorded. Results indicated suboptimal pre-implantation embryo development. Troubleshooting was initiated and led to the amendment of oocyte vitrification practice for donor oocytes derived from a different group of donors that were vitrified between September 2024 and February 2025, during which a quality control parameter in controlling vitrification solution temperature within a designated range during handling of oocytes was implemented (23 ± 2°C). The outcomes of oocyte survival and pre-implantation embryo development were also recorded following the intervention. A retrospective analysis was then performed from the data, comparing outcomes across the two different vitrification strategies (controlled and uncontrolled vitrification solution temperature), to evaluate their impact on oocyte viability and subsequent embryo development. The study received ethical approval from Cyprus International University (EKK24-25/06/10). All participants provided written informed consent for the donation of their oocytes. A total of 170 mature metaphase II (MII) oocytes, donated from 16 consenting donors across 16 donation cycles, were included. These consisted of a controlled solution temperature group (6 donors, *n* = 83 oocytes) and an uncontrolled temperature group (10 donors, *n* = 87 oocytes).

In both groups, solution and room temperatures were monitored using the specified and validated UNI-T UT322 thermometer with the appropriate sensors as described above. In the controlled group, although ambient room conditions were within the recommended range of 23 ± 2°C (Butler *et al.* 2013, Sjoblom & Liperis 2019), the heated stage of the laminar flow hood for the purpose of oocyte vitrification was increased to 29.5°C to establish vitrification solution drop measurement within a room temperature range (23 ± 2°C). The temperature of the vitrification solution was measured immediately before the addition of the oocyte into a mock drop next to the drop used for vitrification. All other parameters, including the type of dish, media volume, and dish placement, were kept consistent throughout the procedures during the study period to ensure stable and representative temperature conditions at the time of oocyte exposure. In addition, all oocyte culture, vitrification, and warming protocols, as well as subsequent embryo culture conditions including the specific brands of materials used were identical across groups. Noted differences were only recorded for media lot numbers between the two study periods. Throughout the duration of the study, only one senior embryologist was responsible for oocyte vitrification and warming. The senior embryologist has had extensive experience in both protocols with staff competency in oocyte vitrification and warming assessed regularly, to ensure consistent quality and adherence to best practices.

### Oocyte donors

All participants in the study were anonymous individuals, with ages ranging from 20 to 30 years old at the time of oocyte donation. The candidates underwent thorough screening, including serological testing for hepatitis B surface antigen, hepatitis C virus, and HIV antibodies, as well as genetic testing for thalassaemia, cystic fibrosis, and chromosomal abnormalities via karyotype analysis. Furthermore, comprehensive evaluations of familial histories concerning genetically inherited diseases were conducted. The controlled ovarian stimulation protocol was performed as previously described by Khurana *et al.* 2022. Following a baseline transvaginal ultrasound assessment on the second day of menstruation, ovarian stimulation was initiated using recombinant follicle-stimulating hormone (rFSH) at doses ranging from 200 to 225 IU per day (Gonal-F, Merck, Germany). The initial dosage was individualised based on the patient’s age, antral follicle count (AFC), body mass index (BMI), and, if applicable, previous responses to ovarian stimulation. Concurrently, a fixed dose of medroxyprogesterone acetate (MPA) (5 mg tablet, Deva, Turkey) was administered at 10 mg once daily, beginning on the second day of menstruation and continuing until the day of ovulation trigger. Upon the achievement of at least three follicles measuring 18 mm in diameter, patients were administered 0.2 mg of triptorelin (Gonapeptyl, Ferring), with oocyte retrieval scheduled to occur 35 h subsequent to the injection.

### Oocyte pick-up, denudation, semen preparation, ICSI, and embryo culture

Oocyte aspiration was performed 35 h after HCG administration under ultrasound guidance. Oocytes were washed with handling medium-complete (MHM-C; FuJIFILM Irvine Scientific, USA) containing gentamicin and human serum albumin (HSA), and collected into pre-equilibrated 750 μL drops of continuous single culture-NX Complete (CSCM-NXM) supplemented with Gentamicin and HSA, which was kept under humidified and heated conditions (at 37°C, 6% CO_2_ and 5% O_2_) in a benchtop incubator (G210 InviCell From K-Systems™) for 2 h until denudation. Enzymatic removal of cumulus cells was performed using 80 IU/mL hyaluronidase (FUJIFILM Irvine Scientific, USA). Following denudation, intracytoplasmic sperm injection (ICSI) was performed in multipurpose handling medium-complete (MHM-C; FUJIFILM Irvine Scientific, USA) containing Gentamicin and HSA and microinjected oocytes were cultured individually in a special pre-equilibrated culture dish (EmbryoSlide, Vitrolife) for embryo development. In this study, only continuous single culture-NX Complete (CSCM-NXM) supplemented with Gentamicin and HSA was used for embryo culture. EmbryoSlide wells were filled with 25–30 μL CSCM-NXM and covered with 1.4 mL Heavy oil (Kitazato) to prevent evaporation and equilibrated overnight before use. All oocytes/embryos were cultured in a time-lapse incubator (EmbryoScope, Vitrolife, Sweden) at 37°C, 6% CO_2_, and 5% O_2_. The sperm used for fertilisation was derived from donors and was obtained from a commercial sperm bank (Cryos International, Denmark), with each sample being thawed and subsequently subjected to two washing cycles utilising a sperm washing medium, specifically a modified HTF medium supplemented with HSA at a concentration of 5.0 mg/mL (FUJIFILM Irvine Scientific, USA).

### Oocyte vitrification and warming procedure

The study was conducted in a controlled laboratory environment. The room temperature and vitrification solution temperatures were recorded using the UNI-T UT322 thermometer, and validated with an NIST-traceable mercury-calibrated thermometer as described above. The Vitrification Freeze/Warm Kit (Vit Kit-Freeze NX/Vit Kit-Warm NX; FUJIFILM Irvine Scientific, USA) was employed throughout the course of this investigation. The procedures for the vitrification and warming of oocytes were executed in accordance with the specifications provided by the manufacturers. The Cryotop® carrier device (Kitazato, Japan) and Nunc IVF dish 90 mm, non-treated (Thermo Scientific, USA), were used for the vitrification processes. In brief, Equilibration NX–ES constitutes a dual-buffered solution (HEPES & MOPS) derived from continuous single culture medium (CSCM), which incorporates gentamicin sulphate, 7.5% (v/v) of both DMSO and ethylene glycol, alongside 20% (v/v) of dextran serum supplement (DSS). Oocytes were equilibrated as follows: up to 2 oocytes were placed into one 20 μL drop of WS for 1 min, following to merging the drop to 20 μL drops of ES for 2 min, followed by merging the drop to an additional 20 μL ES for 2 min, and followed by moving the oocytes to a fresh ES for 6–10 min as per manufacturer’s recommendations. Vitrification NX–VS is characterised as a dual-buffered solution (HEPES & MOPS) of CSCM that includes gentamicin sulphate, 15% (v/v) of each DMSO and ethylene glycol, 20% (v/v) DSS, in addition to 0.5 M trehalose. DSS represents a protein supplement composed of 50 mg/mL therapeutic-grade HSA and 20 mg/mL dextran. DSS is incorporated at a concentration of 20% (v/v) in Vit Kit–Freeze NX, yielding a final concentration of 10 mg/mL HSA and 4 mg/mL dextran. For the vitrification step, the oocytes were moved from the ES to a 50 μL drop of VS solution for 60 s before loading them on the cryodevice and submerging them to liquid nitrogen as per manufacturer’s recommendations.

In terms of the warming of the oocytes, the thawing kit contains a thawing solution (TS) that contains 2 mL in each vial of a 1 M sucrose, 20% DSS, and gentamicin composition in an M-199 HEPES buffered medium. Also, the Kit contains a dilution solution (DS) consisting of 2 mL of a composition of 0.5 M sucrose, 20% DSS, and gentamicin in the same M-199 HEPES buffered medium. Finally, the washing solution (WS) containing 2 mL contains 20% DSS and gentamicin in an M-199 HEPES buffered medium. For thawing, the oocytes were submerged in 1 mL of TS solution for 1 min, followed by washing in 50 μL drop of DS solution for 4 min and followed by two separate 50 μL WS drops washes for 4 min each as per manufacturers recommendations. After warming, oocytes were placed in culture media and checked for viability (warmed oocytes were considered not to survive if lysed, highly vacuolised or otherwise impaired in cytoplasmic or extracytoplasmic structures) and incubated under standard conditions at 37°C (6% CO_2_, 5% O_2_) until the ICSI procedure.

### Fertilisation and embryo culture

Upon completion of warming of oocytes, ICSI was conducted in multipurpose handling medium-complete (MHM-C; FUJIFILM Irvine Scientific, USA) containing gentamicin and HSA, with microinjected oocytes cultured individually within a specialised pre-equilibrated culture dish (EmbryoSlide, Vitrolife) to facilitate embryonic development. This investigation exclusively employed continuous single culture-NX Complete (CSCM-NXM) supplemented with gentamicin and HSA for the purpose of embryo culture. The wells of the EmbryoSlide were filled with 25–30 μL CSCM-NXM and subsequently covered with 1.4 mL of heavy oil (Kitazato) to mitigate evaporation, allowing for overnight equilibration prior to application. All embryos were incubated within a time-lapse incubator (EmbryoScope™+, Vitrolife) at 37°C, 6% CO_2_, and 5% O_2_. Fertilisation assessment was conducted 16–18 h post insemination. Embryos were cultured until day 6 of embryo development, and Gardner and Schoolcraft’s criteria for blastocyst grading, in addition to morphokinetic data, were used to evaluate embryo quality (Gardner & Schoolcraft 1999).

### Statistical analysis

Data were analysed using both parametric and non-parametric statistical methods as appropriate. For comparison of continuous variables between controlled and uncontrolled temperature groups, including stimulation days, daily usage, BMI, total usage, age, collected oocytes, and temperature measurements, the Mann–Whitney U test was employed due to the small sample sizes and without assuming normal distribution of the data. Results are presented as mean ± standard deviation (M ± SD) along with 95% confidence intervals (CI).

For categorical variables related to oocyte and embryo quality outcomes, chi-square tests (*χ*^2^) were used to compare proportions between groups, including oocyte survival percentage, fertilisation rate, and blastulation percentage. Fisher’s exact test was applied for comparing pronuclear arrest, post-pronuclear fading zygote arrest, and cleavage arrest percentages between groups, as this test is more appropriate when expected cell frequencies are small. For these arrest parameters, one-sided 97.5% confidence intervals were calculated to reflect the directionality of the observed differences. Statistical significance was set at *P* < 0.05 for all analyses. Statistical analyses were performed using SPSS, version 27.

## Results

### Baseline donor characteristics are comparable between controlled and uncontrolled temperature groups

Baseline characteristics of oocyte donors did not differ significantly between the controlled (6 donors) and uncontrolled (10 donors) temperature groups across all assessed parameters (Table 1). The mean number of stimulation days was comparable between the uncontrolled (10.36 ± 1.36) and controlled (10.67 ± 1.51) groups (*P* = 0.76). Daily gonadotropin doses were similar in the uncontrolled (209.09 ± 16.85 IU) and controlled (208.33 ± 12.91 IU) groups (*P* = 0.88). BMI values were also comparable between groups (22.00 ± 1.00 vs 21.83 ± 0.98; *P* = 0.80). Total gonadotropin consumption during stimulation did not differ between the uncontrolled (2,170.45 ± 224.67 IU) and controlled (2,220.83 ± 334.07 IU) groups (*P* = 0.80). Mean age was comparable (24.64 ± 2.25 vs 25.00 ± 3.03 years; *P* = 0.80). No significant differences in antral follicle count (AFC) were observed between donors in the two groups (25.25 ± 6.1 vs 23.0 ± 1; *P* = 0.6) and the number of oocytes retrieved was similar (22.55 ± 1.92 vs 22.67 ± 2.07; *P* = 0.96).

**Table 1:** Comparison of patient characteristics between controlled and uncontrolled groups.

Parameter	Mean ± SD		MWU test	*P*-value	95% CI	
	Uncontrolled	Controlled			Uncontrolled	Controlled
Stimulation days	10.36 ± 1.36	10.67 ± 1.51	29.5	0.76	9.45, 11.27	9.09, 12.25
Daily usage (IU)	209.09 ± 16.85	208.33 ± 12.91	31	0.88	197.77, 220.41	194.78, 221.88
BMI (kg/m^2^)	22 ± 1	21.83 ± 0.98	30	0.80	21.33, 22.67	20.8, 22.86
Total usage (IU)	2,170.45 ± 224.67	2,220.83 ± 334.07	30	0.80	2,019.52, 2,321.38	1,870.19, 2,571.47
Age (years)	24.64 ± 2.25	25 ± 3.03	30	0.80	23.13, 26.15	21.82, 28.18
Antral follicle count	25.25 ± 6.1	23.0 ± 1.8	34.5	0.6	21.3, 29.1	20.6, 25.3
Number of collected oocytes	22.55 ± 1.92	22.67 ± 2.07	32	0.96	21.26, 23.84	20.5, 24.84

SD, standard deviation; MWU, Mann–Whitney U; CI, confidence interval; BMI, body mass index.

### Significant differences in vitrification medium temperatures between groups

Room temperature was significantly lower in the uncontrolled temperature group (22.82 ± 0.55°C) compared to the controlled group (24.77 ± 0.1°C; Mann–Whitney *U* = 6, *P* < 0.001). A more pronounced difference was observed in the temperature of the oocyte equilibration and vitrification medium (‘drop temperature’), which averaged 19.65 ± 0.43°C under uncontrolled conditions versus 23.85 ± 0.43°C in the controlled setting (Mann–Whitney *U* = 0, *P* < 0.001) (Table 2). Graphical presentation of temperature curves over time for room temperature and vitrification solution temperature for the two groups is shown in Supplementary Fig. 1 (see section on Supplementary materials given at the end of the article). Excluding vitrification, for all other processes of oocyte and embryo handling, heated surface and drop temperature measurements are shown in Supplementary Table 1.

**Table 2:** Comparison of oocyte and embryo quality between uncontrolled group and controlled group conditions. Data are presented as mean ± SD or as %.

Parameter	Uncontrolled	Controlled	Test	*P*-value	95% CI	
					Uncontrolled	Controlled
Room temperature °C	22.85 ± 0.839	24.77 ± 0.09776	MWU = 6	<0.001	22.67, 23.03	24.56, 24.98
VMT °C (drop temperature)	19.65 ± 0.839	23.85 ± 0.3780	MWU = 0	<0.001	19.47, 19.83	23.77, 23.93
MII oocytes, total *n*	87	83	—	—	—	—
Oocyte survival %	80/87	82/83	*χ*^2^ = 3.0387	0.08	0.8412, 0.9670	0.4475, 0.6891
Fertilisation rate %	70/87	69/82	*χ*^2^ = 0.181	0.67	0.7057, 0.8819	0.7442, 0.912
Pronuclear arrest %*	10/70	0/69	Fisher’s exact	<0.001	0.0707, 0.2471	0, 0.0521
Post-pronuclear fading zygote arrest %*	20/70	0/69	Fisher’s exact	<0.001	0.1840, 0.4062	0, 0.0521
Cleavage arrest %* (blocked at cleavage stage)	35/70	0/69	Fisher’s exact	<0.001	0.3780, 0.6220	0, 0.0521
Cleavage rate %*	40/70	69/69	Fisher’s exact	<0.001	0.4475, 0.6891	0.9479, 1
Blastulation %	5/70	56/69	*χ*^2^ = 90.5211	<0.001	0.0236, 0.1589	0.6994, 0.8957

VMT, vitrification medium temperature; MWU, Mann–Whitney U; CI, confidence interval; MII, metaphase II.

* One-sided 97.5% CI was used for these parameters.

### Comparable oocyte survival and fertilisation rates under controlled and uncontrolled conditions

A total of 87 metaphase II (MII) oocytes were exposed to uncontrolled conditions and 83 to controlled conditions. Fertilisation rates were comparable under uncontrolled and controlled conditions (70/87 vs 69/82; *χ*^2^ = 0.18, *P* = 0.67) (Table 2).

Oocyte survival after warming was assessed immediately under an inverted microscope based on morphological criteria. Surviving oocytes were defined as those exhibiting an intact zona pellucida with intact oolemma and oocytes were considered non-surviving if they displayed lysis of the oolemma when examined under the stereo microscope. Oocyte survival following warming did not differ significantly between the two groups (80/87 vs 82/83; *χ*^2^ = 3.04, *P* = 0.08), with overlapping confidence intervals.

### Pronuclear, post-pronuclear fading zygote arrest, and cleavage arrest rates were significantly higher under uncontrolled conditions

Pronuclear arrest was observed in 10/70 zygotes in the uncontrolled group, whereas no arrest occurred in the controlled group (0/69; *P* < 0.001, Fisher’s exact test). Post-pronuclear fading zygote arrest followed a similar pattern, affecting 20/70 oocytes in the uncontrolled group and none in the controlled group (0/69; *P* < 0.001). Cleavage arrest occurred in 35/70 oocytes cultured under uncontrolled conditions, while all zygotes cleaved in the controlled group (69/69; *P* < 0.001). As a result, the overall cleavage rate was significantly reduced in the uncontrolled group (40/70). Blastulation was also markedly impaired, with only 5/70 oocytes reaching the blastocyst stage in the uncontrolled group compared to 56/69 in the controlled group (*χ*^2^ = 90.52, *P* < 0.001) (Table 2). Morphokinetic data comparisons for the two groups are provided in Supplementary Table 2. Comparisons of embryological and clinical outcomes between fresh autologous and donor oocyte cycles from March 2024 to February 2025 are shown in Supplementary Table 3.

## Discussion

This study investigates the impact of vitrification media drop temperature as a potential novel quality control parameter to improve cryopreservation outcomes based on oocyte vitrification practice that was introduced in a new built-for-purpose IVF laboratory. While oocyte survival and fertilisation rates remained unaffected, embryo development was significantly compromised under uncontrolled vitrification solution drop temperatures (∼20°C). Controlled drop temperatures (∼24°C) yielded higher blastulation rates and reduced developmental arrest. Comparable patient characteristics across groups strengthen the possible attribution of these effects to vitrification media drop temperature rather than baseline variability.

In this observational study, cleavage arrest was significantly more frequent in the uncontrolled temperature group (50%) compared to the controlled group (0%), reinforcing the notion that thermal instability can impair embryonic development (Sjoblom & Liperis 2019). Previous studies have shown the importance of precise temperature control during vitrification, as even slight deviations may disrupt oocyte physiology (Elder & Dale 2020). Oocytes are particularly vulnerable to thermal fluctuations (Larman *et al.* 2007, Martinez-Rodero *et al.* 2025). Previous studies have reported that cold-induced spindle disassembly can lead to developmental arrest (Wang *et al.* 2001*b*, Larman *et al.* 2007).

Oocytes are extremely thermosensitive and are particularly vulnerable to cryoinjuries (Zenzes *et al.* 2001). Their vulnerability can be attributed primarily to their considerably large size, high water content (75–85%), and distinctive intracellular architecture, rendering them among the most complex biological entities to successfully undergo freezing procedures (Zenzes *et al.* 2001, Chang *et al.* 2024). Abrupt reductions in temperature can precipitate cold-shock injuries in temperature-sensitive structures, compromising their functionality by modifying membrane permeability and inflicting damage on intracellular organelles, such as the cytoskeleton and meiotic spindle (Larman *et al.* 2007, Chang *et al.* 2024). The extreme vulnerability of oocytes to cryoinjury is largely attributed to the sensitivity of the meiotic spindle to temperature fluctuations (Zenzes *et al.* 2001). During all stages of *in vitro* handling, not only regarding cryopreservation, variations in temperature that fall beneath the physiological range (37.0 ± 0.2°C) can have a direct impact on the developmental capacity of oocytes, resulting in disturbances in cytoskeletal structures, particularly the depolymerisation of the meiotic spindle (Almeida & Bolton 1995, Sjoblom & Liperis 2019). About 10–30 min exposure to temperatures near 20°C can disrupt spindle integrity, significantly impairing subsequent embryonic development after fertilisation (Almeida & Bolton 1995). At 0°C, rapid depolymerisation occurs, leading to irreversible spindle disruption (Zenzes *et al.* 2001). While cryoprotectants help preserve spindle structure during freezing, the warming process often results in severe compromise (Zenzes *et al.* 2001). However, studies have shown that the spindle can reassemble post-cryopreservation and acquire correct chromosome alignment and segregation following fertilisation (Zenzes *et al.* 2001, Koutlaki *et al.* 2006, Martínez-Burgos *et al.* 2011, Tamura *et al.* 2013, Sjoblom & Liperis 2019, Liperis & Sjöblom 2023, Pantos *et al.* 2024, Martinez-Rodero *et al.* 2025).

Osmotic shock, arising from abrupt alterations in osmolarity during the addition or removal of cryoprotectant agents (CPAs), poses a risk to cellular membranes and diminishes oocyte viability (Chang *et al.* 2024). The manipulation of temperature, CPA concentration, and exposure duration must be meticulously calibrated to achieve a harmonious equilibrium between cryoprotection and cellular integrity (Chang *et al.* 2024, Martinez-Rodero *et al.* 2025). One of the most important parameters during oocyte vitrification is temperature (Elder & Dale 2020). Elevated temperatures augment the kinetic energy and consequently the motility of the molecules, thereby enhancing the diffusion rate (Chang *et al.* 2024). Given that the processes of loading and unloading permeable CPAs are entirely contingent upon the diffusion rate, an increase in the temperature employed for oocyte vitrification correlates with a reduction in the duration required for the loading and unloading of CPAs (Chang *et al.* 2024, Stachecki 2024).

In accordance with the majority of protocols from commercially available kits for oocyte vitrification, it is customary to utilise room temperature (22–25°C) as a reference temperature for the loading and unloading of CPAs across the oocyte membrane due to its procedural simplicity (Chang *et al.* 2024, Martinez-Rodero *et al.* 2025). Routine quality control procedures in most IVF laboratories include temperature monitoring of the heated stage, ensuring that media remain at 37 ± 0.2°C using validated instruments. However, less attention is typically paid to the temperature of cryopreservation solutions during handling on non-heated stages. It remains to be established whether controlling vitrification drop temperature can improve vitrification protocols in terms of overall cryopreservation outcomes.

Current commercially available vitrification kits typically specify room temperature (22–27°C) for CPA loading and unloading; however, these protocols often lack adequate control over the vitrification media temperature (Elder & Dale 2020, Stachecki 2024). Every laboratory operates under different conditions. Laboratory factors such as room temperature, air velocity within the laminar flow hood, and dish type may inadvertently lower the temperature of the vitrification solution, potentially affecting the efficiency and consistency of cryopreservation (Sjoblom & Liperis 2019, Elder & Dale 2020, Liperis & Sjöblom 2023).

Recent studies investigating modified CPA equilibration strategies at different temperatures provide further context for these findings. Martinez-Rodero *et al.* (2025) reported that controlled CPA exposure conditions, including temperature management during equilibration, can enhance developmental competence following vitrification, supporting the concept that subtle procedural refinements may yield measurable improvements in downstream embryo outcomes (Martinez-Rodero *et al.* 2025). Similarly, Liu *et al.* demonstrated that temperature-dependent equilibration protocols influence cellular osmotic responses and CPA permeability, highlighting the importance of optimising both exposure conditions and thermal environment to minimise cryoinjury (Liu *et al.* 2017). Together, these studies reinforce the biological plausibility that even modest deviations in solution temperature during CPA handling may alter intracellular CPA dynamics, osmotic stress, and cytoskeletal stability, ultimately affecting post-warming developmental potential. Our results extend this body of evidence by suggesting that not only equilibration temperature per se but also the temperature stability of vitrification solution droplets during routine laboratory handling may represent a previously under-recognised determinant of embryo development.

Our findings, upon verification, will highlight the importance of continuously monitoring and regulating vitrification media temperature to ensure optimal cryopreservation outcomes. Published benchmarks and key performance indicators (KPIs) for cryopreservation offer crucial insights for evaluating procedural effectiveness and results (Medicine 2012). Nevertheless, these benchmarks might not completely reflect the advancements in techniques and the introduction of new quality control measures. The Alpha consensus expert assembly has delineated 14 KPIs for cryopreservation; however, it did not provide specific guidance on particular cryopreservation techniques or equipment (Medicine 2012). Importantly, the consensus has not taken into consideration the possible impact of solution temperature regulation on vitrification outcomes. Considering that fluctuations in oocyte vitrification solution temperature can significantly affect downstream developmental capacity as shown from the outcomes of this study, this aspect represents a neglected quality control measure. The lack of standardised vitrification solution temperature monitoring in current protocols may lead to inconsistencies in results. By integrating solution temperature as an additional quality control criterion, existing KPIs could be enhanced, potentially increasing the dependability of vitrification success rates. Furthermore, even the vitrification protocol manuals issued by commercial firms, which contain specifications from the manufacturers, very commonly use room temperature with no mention of the working vitrification medium solution temperature (Medicine 2012, Campbell & Barrie 2024).

Despite the fact that our investigation yielded significant insights, some limitations must be acknowledged, including the observational nature and asynchronous design of the study which inherently limits the ability to draw causal conclusions, as well as the small sample size and limited number of donors, which constrain both the statistical power and the generalisability of the findings. In addition, since donors were recruited at different time frames, there is a possibility that the outcomes reflected biological variability between the donors, even though there were no significant differences between background characteristics and all recruited donors had proven parity. Furthermore, potential confounding factors should be considered, particularly the fact that the observations were made in a newly established laboratory setting. A prospective investigation using sibling oocytes to control for donor-related factors would offer a more robust framework, but raises ethical concerns that preclude its implementation. Further experiments using animal models could help determine whether the findings of this study are specifically attributable to the altered drop temperature of the vitrification solution. The use of apoptotic biomarkers, such as caspase-3, in addition to developmental parameters, can be explored. The oocytes in this study were obtained from young, healthy donors, which may not accurately represent the wider patient demographic, including the elderly and those exhibiting diminished ovarian reserve and other infertility causes. In addition, although our study demonstrates the possibility that temperature stability is important, the exact optimal temperature range for vitrification solutions will need to be determined. In conclusion, this study suggests that temperature may influence oocyte developmental outcomes following fertilisation, but this is not conclusively shown here due to the many confounding variables that have not been assessed. Controlled vitrification solution drop temperature shows improved and not compromised embryo development, while uncontrolled temperatures can potentially compromise blastulation rates and increase developmental arrest. Regardless of room temperature, the media temperature potentially plays a more crucial role in achieving successful oocyte cryopreservation outcomes. By identifying the importance of temperature stability as a novel quality control parameter, we can refine vitrification protocols and improve cryopreservation outcomes.

## Supplementary materials





## Declaration of interest

G Liperis and M Serdarogullari are Associate Editors of *Reproduction *&* Fertility* and were not involved in the peer review or editorial process for this paper, on which they are listed as authors. All authors report no conflicts of interest directly or indirectly related to this work submitted for publication.

## Funding

This research did not receive any specific grant from any funding agency in the public, commercial, or not-for-profit sector.

## Author contribution statement

MS, ZA, GR, GL, and MM conceived the study. MS, ZA, and YY collected the data. MS, ZY, GR, and OA performed formal analysis. MS, ZA, GR, MM, GL, GR, OA, and BY designed the methodology. MS, GL, GR, OA, ZA, and ZY wrote, reviewed, and edited the manuscript. MM and GL supervised the study. All authors reviewed the results and approved the final version of the manuscript.
