# Supplementary material for: Media temperature control: a potentially important quality control parameter in human oocyte vitrification
Source: Reprod Fertil. 2026 May 15;7(2):RAF260003. doi: 10.1530/RAF-26-0003 (PMC13193071; doi:10.1530/RAF-26-0003)
Supplement: Supplementary file 1 [file supplementary_figure.pptx]

## Slide 1
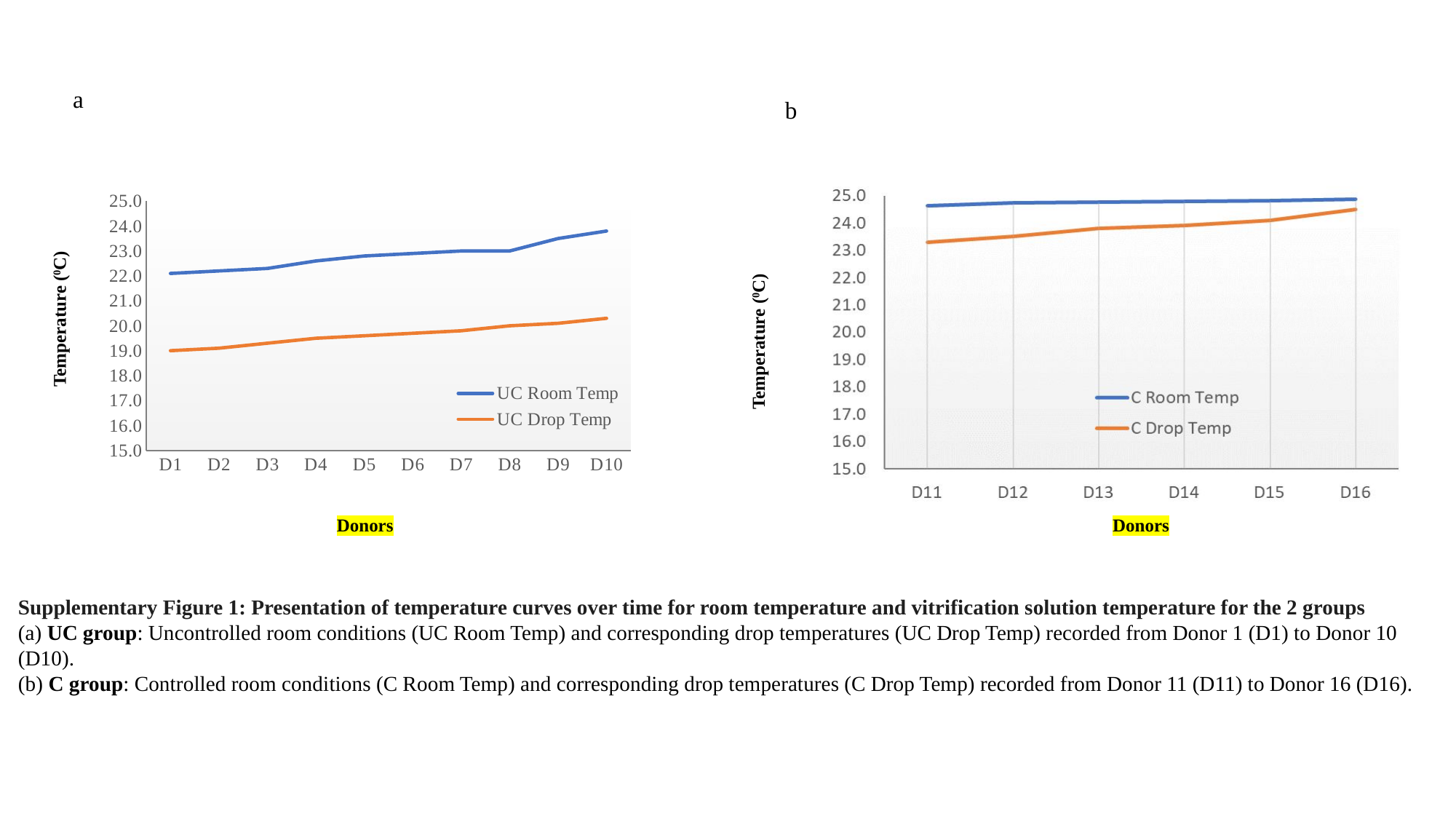

a
b
### Chart
| Category | UC Room Temp | UC Drop Temp |
|---|---|---|
| D1 | 22.1 | 19.0 |
| D2 | 22.2 | 19.1 |
| D3 | 22.3 | 19.3 |
| D4 | 22.6 | 19.5 |
| D5 | 22.8 | 19.6 |
| D6 | 22.9 | 19.7 |
| D7 | 23.0 | 19.8 |
| D8 | 23.0 | 20.0 |
| D9 | 23.5 | 20.1 |
| D10 | 23.8 | 20.3 |
Temperature (0C)
Temperature (0C)
Donors
Donors
Supplementary Figure 1: Presentation of temperature curves over time for room temperature and vitrification solution temperature for the 2 groups
(a) UC group: Uncontrolled room conditions (UC Room Temp) and corresponding drop temperatures (UC Drop Temp) recorded from Donor 1 (D1) to Donor 10 (D10).
(b) C group: Controlled room conditions (C Room Temp) and corresponding drop temperatures (C Drop Temp) recorded from Donor 11 (D11) to Donor 16 (D16).
