# Supplementary material for: Media temperature control: a potentially important quality control parameter in human oocyte vitrification
Source: Reprod Fertil. 2026 May 15;7(2):RAF260003. doi: 10.1530/RAF-26-0003 (PMC13193071; doi:10.1530/RAF-26-0003)
Supplement: Supplementary file 2 [file supplementary_tables.pdf]

Supplementary Table 1: Processes of oocyte and embryo handling, heated surface and drop temperature measurements

	ICSI Rig Display Temperature °C	ICSI Rig Surface Temperature °C	ICSI Rig Drop Temperature °C	Laminar Flow-1Display Temperature °C	Laminar Flow-1 Surface Temperature °C	Laminar Flow-1 Solution Temperature °C	Laminar Flow-2 Display Temperature °C	Laminar Flow-2 Surface Temperature °C	Laminar Flow-2 Solution Temperature °C
March 2024	37±0	37±0	36.95±01	37.13±0.08	36.98±0.5	37±0.2	39±0	38.27±0.22	37±0.2
April 2024	37.01±0.3	37±0	36.87±0.1	37.25±0.14	37.14±0.22	37±0.2	39±0	38.34±0.25	37±0.2
May 2024	37.01±0.3	37±0	36.87±0.1	37.25±0.14	37.14±0.22	37±0.2	39±0	38.36±0.26	37±0.2
June 2024	37±0	37±0	36.84±01	37.2±0.28	36.88±0.13	37±0.2	39±0	38.44±0.3	37±0.2
July 2024	37±0	37±0	36.88±0.1	37.05±0.09	37.14±0.11	37±0.2	39±0	38.51±0.1	37±0.2
August 2024	37±0	37±0	36.86±0.1	37.2±0	37.47±0.17	37±0.2	39±0	38.81±0.05	37±0.2
September 2024	37±0	37±0	36.92 ±0.1	37.2±0	37.71±0.20	37±0.2	39±0	38.60±0.16	37±0.2
October 2024	37±0	37±0	36.8±0.1	37.2±0	37.50±0.22	37±0.2	39±0	38.62±0.12	37±0.2
November 2024	37±0	37±0	37.03±0.3	37.2±0	37.73±0.08	37±0.2	39±0	38.73±0.17	37±0.2
December 2024	37±0	37±0	37.03±04	37.2±0	37.73±0.36	37±0.2	39±0	38.52±0.18	37±0.2
January 2025	37±0	37±0	36.9±01	37.2±0	37.11±0.19	37±0.2	37.08±0.14	38.44±0.18	37±0.2
February 2025	37±0	37±0	36.9±01	37.2±0	37.53±0.08	37±0.2	38±0	38.27±0.07	37±0.2

Values are presented as mean ± standard deviation

Supplementary Table 2: Comparison of embryo morphokinetics between the uncontrolled and controlled groups.

Embryo	Uncontrolled Group	Controlled Group	p-value

**morphokinetics**			
tPNa	10.35 (9.06-11.78) (n=70)	8.70 (7.70-9.90) (n=69)	<0.001
tPNf	29.25 (24.35-33.68) (n=60)	24.60 (22.40-26.30) (n=69)	<0.001
t2	33.50 (28.80-37.02) (n=40)	27.30 (25.10-28.60) (n=69)	<0.001
t3	38.20 (35.50-42.80) (n=39)	37.80 (34.90-40.70) (n=69)	0.377
t4	48.00 (43.00-52.60) (n=37)	40.70 (37.30-44.70) (n=69)	0.002
t5	52.75 (48.00-59.02) (n=32)	50.60 (46.00-56.20) (n=69)	0.226
t6	52.70 (48.23-62.42) (n=28)	52.60 (48.80-59.00) (n=69)	0.799
t7	60.90 (52.08-75.97) (n=26)	56.40 (51.50-63.50) (n=69)	0.409
t8	63.05 (53.12-86.15) (n=24)	61.20 (54.50-69.70) (n=69)	0.429
t9+	71.80 (61.50-86.60) (n=23)	71.60 (67.00-80.40) (n=69)	1
tM	98.20 (90.60-100.50) (n=5)	88.40 (81.20-93.40) (n=69)	0.286
tSB	111.10 ± 16.57 (n=5)	99.22 ± 6.34 (n=56)	0.185
tB	122.22 ± 20.98 (n=5)	106.63 ± 6.69 (n=56)	0.172
tEB	123.45 (108.10-140.28) (n=4)	110.60 (104.9-116.6) (n=54)	0.373

Values are presented as median (interquartile range) or mean ± standard deviation, according to distribution. Time points are expressed in hours post-insemination. P-values reflect comparisons between groups using the Mann–Whitney U test or independent t-test, as appropriate. Abbreviations: tPNa – time of pronuclei appearance; tPNf – time of pronuclei fading; t2–t9+ – time to 2-cell to 9+ cell stage; tM – time of morula formation; tSB – time of start of blastulation; tB – time of full blastocyst formation; tEB – time of expanded blastocyst formation.

Supplementary Table 3: Comparison of clinical and embryological outcomes between autologous and donor oocyte cycles during the study period, before and after implementation of the vitrification temperature control.

**March 2024-February 2025**	**March 2024-August 2024 Fresh Autologous Cycle**	**September 2024- February 2025 Fresh Autologous**	**p-value**	**Fresh Donor Oocyte Cycle March 2024-August 2024**	**Fresh Donor Oocyte Cycle September 2024-**	**p-value**

		Cycle			February 2025	
**Number of Patients**	24	124		47	42	
**Female Age (Mean±Std)**	36.7±5.7	34.7±5.2	0.11	26.1±2.4	26.6±2.1	0.29
**Maturation rate (%)**	76	83.8	0.35	80.2	80.3	0.99
**Fertilisation Rate (%)**	76.6	86.5	0.21	82	77.5	0.59
**Total blastulation Rate (%)**	61.6	61.6	1	72.6	69.6	0.75
**Usable Blastocyst Rate (%)**	54.8	50.8	0.71	62.8	61.8	0.92
**Euploidy rate Rate (%)**	60.9	60.9	1	75	74.6	0.96
**Pregnancy Total Rate (%)**	72.7	84.2	0.17	80.7	74.4	0.47
**Pregnancy Single Embryo Transfer Rate(%)**	60	92.3	<0.001	72.7	73.9	0.89
**Pregnancy Double Embryo Transfer Rate (%)**	83.3	66.6	0.10	85	100	.00089
**Fresh Embryo Transfer Pregnancy Rate (%)**	33.3	100	<0.001	55.6	100	<0.001
**Frozen Embryo Transfer Pregnancy Rate (%)**	87.5	82.4	0.54	90.9	70.6	0.01

Values are presented as means ± standard deviation or percentages, as appropriate. Statistical comparisons between own oocyte and donor oocyte cycles were performed using the independent t-test or chi-square test. P-values <0.05 were considered statistically significant.
